# Event and Comment

**Published:** 1923-05

**Authors:** 


### DENTAL REGISTER

THE MAGAZINE OF DENTISTRY

Vol. LXXVII MAY, 1923 No. 5

### EVENT AND COMMENT

BY VOTRE AMI DENTAIRE

One of the recent developments of the scientific explanation of spirit pictures is that there are visions which the eye can not see, just as there are sounds that the ear can not hear. However, the delicately sensitized photographic plate is sensitive to these spirit pictures or the energy radiating from same and the delicate chemicals are changed, thereby producing a photograph. This whole subject is not yet very clear at all, but one must admit that all great discoveries are vague to the lay mind at first and the expounder and supporter of the inexplainable theory is generally looked upon with skepticism. Columbus had no easy path in putting over his grand theory about the world being round. What is past looks easy, that ahead of us looks difficult, and one of the subjects is spirit pictures. Those who believe in them feel the truth by intuition rather than by scientific theory and thus at the present time the public lacks the connecting scientific reasoning without which the subject lacks logical explanation.

The practice of prophylaxis is becoming a fascinating specialty and a paying one too. Preventive dentistry, prophylaxis, is a branch of dentistry which enables the practitioner to save the teeth and that is the prime object of dentistry. The patient can be called back for examination and prophylaxis three or four times a year and the dentist does not have to wait until trouble comes, for the object is to prevent trouble.

